# Development and Assessment of a Point-of-Care Application (Genomic Medicine Guidance) for Heritable Thoracic Aortic Disease

**DOI:** 10.2196/55903

**Published:** 2024-10-08

**Authors:** Rohan Patil, Fatima Ashraf, Samer Abu Dayeh, Siddharth K Prakash

**Affiliations:** 1 McGovern Medical School, University of Texas Health Science Center at Houston, Houston, TX, United States; 2 McWilliams School of Bioinformatics, University of Texas Health Science Center at Houston, Houston, TX, United States; 3 Department of Internal Medicine, John P and Kathrine G McGovern Medical School, University of Texas Health Science Center at Houston, Houston, TX, United States

**Keywords:** genomic medicine, point of care, thoracic aortic aneurysm, aortic dissection, decision support

## Abstract

**Background:**

Genetic testing can determine familial and personal risks for heritable thoracic aortic aneurysms and dissections (TAD). The 2022 American College of Cardiology/American Heart Association guidelines for TAD recommend management decisions based on the specific gene mutation. However, many clinicians lack sufficient comfort or insight to integrate genetic information into clinical practice.

**Objective:**

We therefore developed the Genomic Medicine Guidance (GMG) application, an interactive point-of-care tool to inform clinicians and patients about TAD diagnosis, treatment, and surveillance. GMG is a REDCap-based application that combines publicly available genetic data and clinical recommendations based on the TAD guidelines into one translational education tool.

**Methods:**

TAD genetic information in GMG was sourced from the Montalcino Aortic Consortium, a worldwide collaboration of TAD centers of excellence, and the National Institutes of Health genetic repositories ClinVar and ClinGen.

**Results:**

The application streamlines data on the 13 most frequently mutated TAD genes with 2286 unique pathogenic mutations that cause TAD so that users receive comprehensive recommendations for diagnostic testing, imaging, surveillance, medical therapy, and preventative surgical repair, as well as guidance for exercise safety and management during pregnancy. The application output can be displayed in a clinician view or exported as an informative pamphlet in a patient-friendly format.

**Conclusions:**

The overall goal of the GMG application is to make genomic medicine more accessible to clinicians and patients while serving as a unifying platform for research. We anticipate that these features will be catalysts for collaborative projects aiming to understand the spectrum of genetic variants contributing to TAD.

## Introduction

The global incidence of thoracic aortic dissections (3-6 cases per 100,000 person-years) is almost certainly underestimated because up to half of individuals who experience a dissection die before reaching a hospital [1]. In 2022, 7% of out-of-hospital deaths were caused by acute aortic dissections. In more than 20% of cases, a single genetic mutation that is frequently inherited can cause thoracic aortic aneurysms and dissections (TAD) [2]. Genetic testing for TAD can lead to earlier recognition of patients who are at risk for acute aortic dissections and timely individualized interventions that can prevent deaths, such as titration of medical therapies or preventative surgical repair of the aorta. Technological advances have made genetic testing more practical for clinical use [3,4]. Gene-based management of TAD was codified in the recent American College of Cardiology (ACC)/American Heart Association (AHA) guidelines for aortic disease, which provide different recommendations for diagnosis, surveillance, and preventative aortic surgery depending on the mutated gene [5].

However, numerous barriers exist to implementing genetic information into routine clinical workflows [2]. Genetic counseling services are not readily accessible to clinicians or patients. Many clinicians are not prepared to integrate genetic test results into clinical decision-making. In a recent survey, more than 75% of cardiovascular genetic tests were ordered by providers who did not have formal training in clinical genetics [6]. Due to these gaps in care, patients frequently do not receive appropriate pre- or posttest counseling to understand test results or adhere to guideline-based recommendations [7].

Point-of-care tools synthesize complex medical information to guide clinical decision-making [8]. In genomic medicine, point-of-care guidance can lead to more frequent and effective use of genetic information in clinical practice [9-11]. To address genomic medicine care gaps related to TAD, we created Genomic Medicine Guidance (GMG), a point-of-care application that consolidates and clarifies evidence-based recommendations for the clinical management of TAD based on genetic test results. Users who select a pathogenic mutation receive a comprehensive report that describes associated clinical features and recommendations for diagnosis, surveillance, medical therapies, and interventions. We illustrate how the features of the GMG application can lower barriers to the integration of genomic concepts into clinical practice and improve the recognition and treatment of TAD.

## Methods

### Project Design

The GMG application was implemented in a version of REDCap that is hosted at the McWilliams School of Biomedical Informatics at The University of Texas Health Science Center at Houston (UTHealth Houston). REDCap is a secure web-based application designed to support data capture for research studies, providing an intuitive interface for validated data entry, audit trails for tracking data manipulation and export procedures, automated export procedures for seamless data downloads to common statistical packages, procedures for importing data from external sources, and a MySQL database server to facilitate data queries and retrieval. Curated variant information and clinical recommendations are stored in the MySQL database. The user interface is implemented in PHP, a robust and widely used scripting language. The database can be updated continuously by users who enter new variant data through the interface or by project managers who can upload bulk variant data using the data import tools in REDCap.

### Application Code

The GMG application is a custom PHP plug-in that interacts with the REDCap framework to display, extract, add to, or modify the SQL database. The plug-in sequentially queries the SQL database for gene and variant information or associated clinical content in response to user queries. The GMG plug-in is rendered in HTML, JavaScript (JS), and Cascading Style Sheets (CSS). In particular, the application makes use of jQuery, a JS library. When a user directly interfaces with the JS selection fields, Ajax methods in jQuery are leveraged to dynamically load data in the background and update the display without reloading the entire web page. Ajax methods make use of built-in PHP developer functions in REDCap to query the database and return the appropriate information.

The tooltip class is used to display additional information about predefined terms such as acronyms when the user hovers the mouse pointer over text. Results can be formatted for viewing on different screens (phone, desktop, iPad) using the Bootstrap v5.1.2 framework for CSS. We created download and print functions by customizing a PHP script that retrieves data from the SQL database and converts the data to PDF format using the dompdf package. The results file can be emailed to users using built-in REDCap functions.

### User Feedback

We implemented a Qualtrics survey for users to rate GMG in several categories, including usability, clarity, and educational content. The survey included free-response questions that invited users to discuss positive features and areas for improvement. From January to July 2023, the survey was sent to specialist clinicians, general cardiovascular clinicians, clinical geneticists, genetic counselors, and nurses.

### Efficacy Test

Nongenetic clinicians were provided with a sample genetic test report and instructions about how to access GMG. They completed a short survey that required them to retrieve information from GMG about the genetic variants in the sample report (Multimedia Appendix 1). The sample report included two variants of interest: *ACTA2* c.536 G>A (p.Arg179His), a pathogenic variant, and *FBN1* c.3409 C>T (p.Arg1137Cys), a variant of uncertain significance.

### Ethical Considerations

No ethical review was applied for because this study did not involve medical records, patient information, observations of public behaviors, or secondary data analyses. The relevant institutional policies related to these exemptions from review may be found at [12].

## Results

### GMG Data Structure

GMG data fields consist of the gene name, variant information, evidence for pathogenicity, associated clinical features, gene-specific diagnostic workup, variant-specific diagnostic workup, gene-specific surveillance, variant-specific surveillance, gene-specific medical therapy recommendations, gene-specific aortic intervention thresholds, guidance during pregnancy, and activity recommendations. New or revised data can be imported in batches using a spreadsheet template and data import functions in REDCap. All GMG data is stored on an encrypted server with terabytes of storage that is only accessible to members of the study team.

### GMG Genetic Content

The GMG application currently includes data on 2270 pathogenic and likely pathogenic variants in 13 TAD genes: *ACTA2*, *PRKG1*, *TGFBR1*, *TGFBR2*, *SMAD3*, *MYLK*, *TGFB2*, *MYH11*, *FOXE3*, *LOX*, *MFAP5*, *COL3A1*, and *FBN1* (Table 1). The genetic content of GMG is derived from the Montalcino Aortic Consortium, a worldwide collaboration of TAD investigators, and ClinVar, a publicly available National Institutes of Health genetic repository [13,14].

**Table 1.** Current distribution of curated gene variants in the Genomic Medicine Guidance application.

Gene	Variants, n
*FBN1*	1777
*SMAD3*	110
*COL3A1*	99
*TGFBR2*	84
*TGFB2*	64
*TGFBR1*	36
*MYLK*	29
*MYH11*	23
*ACTA2*	21
*LOX*	20
*MFAP5*	3
*FOXE3*	2
*PRKG1*	2

### User Workflow

**Figure 1.**
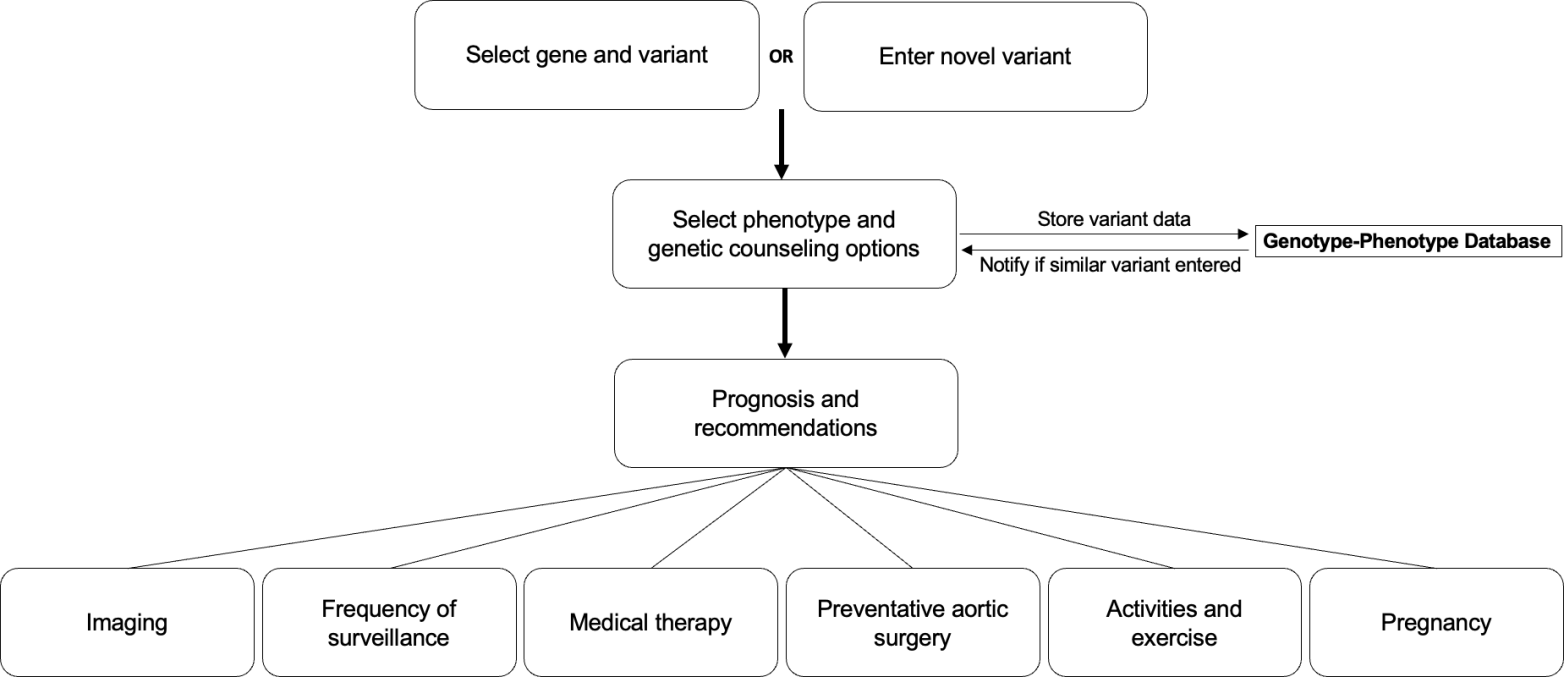
Genomic Medicine Guidance application workflows. Users may select from a list of curated variants or enter a new variant in one of 13 TAD genes that cause thoracic aortic aneurysms or thoracic aortic dissections. The application is available at [15].

Users may freely access GMG at [15]. To facilitate point-of-care queries, the home page provides a prompt to select one TAD gene and one variant from curated drop-down lists (Figure 1). For additional guidance, users may view sample test report forms from commercial genetic laboratories with highlighted variant information. After variant selection, users are required to verify the clinical features that prompted the genetic test and to attest that appropriate pretest genetic counseling was provided. If users enter a variant that is not on the curated list, the application interface provides an option for them to upload the variant data and associated phenotypic information. The output tab describes the selected variant, including evidence for pathogenicity in ClinVar; clinical features associated with the variant, including lifetime risks for aortic surgery or dissection; and evidence-based clinical recommendations for diagnostic and surveillance imaging of the aorta, medical therapy, and size thresholds for elective aortic surgery. These recommendations are based on the 2022 ACC/AHA Guidelines for the Diagnosis and Management of Aortic Disease. As future guidelines are published, the clinical recommendations in the application will be regularly updated to reflect new developments. GMG displays clinician and patient outputs that are optimized for mobile viewing and can be printed, downloaded, or emailed in PDF format. The patient education content of GMG includes links to support groups, genetic counseling resources, and advocacy organizations.

### Researcher Workflow

Researchers can enter new data into the GMG application or update current application data using REDCap data import tools. Batch genetic and clinical data for multiple gene variants can be uploaded simultaneously into the GMG genotype-phenotype database using a single data import template spreadsheet.

### Genetic Counseling Content

GMG application users are required to verify that patients were appropriately selected for genetic testing and received counseling about the benefits, risks, and implications of genetic testing. Users can also access genetic counseling resources from within the application, including guidance to locate a genetic counselor for telehealth consultation. Additional links to the Marfan Foundation and the Montalcino Aortic Consortium, two advocacy groups with resources for newly diagnosed patients with TAD, are provided on the output page.

### User Reviews

A total of 38 GMG users completed a feedback survey about their experiences with the application that was created using a customized version of Qualtrics XM. Users uniformly rated four components of the GMG application as very easy to use: the graphical interface (85/100), selection of a curated gene variant (87/100), understanding clinical recommendations (86/100), and entering a new clinical variant (89/100). Users also rated the clinical recommendations as extremely helpful for clinical guidance (90/100) and highly likely to influence their clinical practice (86/100). Users wrote that some genetic and clinical data in the application may be challenging for individuals who do not have formal training in genetics. They also submitted design recommendations to increase user engagement. Other suggestions included outreach to clinics and professional organizations to establish a user network and an instructional video to orient new users.

### Test of Efficacy

Ten users without any formal training in genetics completed the efficacy exercise and survey. Most users (n=7, 70%) correctly determined the most likely probability of aortic events for individuals with the *ACTA2* pathogenic variant. Most users (n=6, 60%) also identified distinctive features that may occur in individuals with this variant, the threshold for elective aortic repair that is recommended for patients with this variant according to current clinical guidelines (n=6, 60%), and diagnostic tests that are recommended for a patient’s workup with this genetic test result (n=7, 70%). The survey also disclosed some knowledge gaps. Fewer than half of the participants correctly distinguished between the clinical significance of the pathogenic variant and the variant of uncertain significance.

## Discussion

Thoracic aortic aneurysms predispose to deadly aortic dissections without obvious physical signs or symptoms and are frequently inherited as single gene mutations. Genetic testing for TAD can identify affected relatives and save lives but is underused because genetic services are not available to many families who are at risk. In this report, we describe a new point-of-care application, GMG, that is intended to expand access to genetic information about genetic cardiovascular diseases including TAD. As reflected by user ratings and the efficacy test results, GMG simplifies the interpretation of genetic data for clinicians who do not have genetic expertise and provides valued clinical guidance based on the test result.

A review of 20 clinical decision support tools for genomic medicine concluded that they improve risk assessment and lead to meaningful changes in clinical decisions [9,11]. For example, one point-of-care application aggregates data on somatic mutations in tumors to guide cancer therapies based on gene-specific prognosis [10]. The Electronic Medical Records and Genomics network developed an application that integrates contextual decision support into electronic health records to streamline access to genetic testing [16].

GMG includes a modular and scalable genotype-phenotype database that can promote collaboration by connecting providers who enter similar genetic variants to resolve variants of uncertain significance or build case series to elucidate new disease phenotypes. Future versions of GMG will leverage existing partnerships with cardiovascular specialists and the CardioGenomic Testing Alliance to incorporate gene-based care guidance for other adult-onset genetic cardiovascular diseases that are primarily managed by nonexpert clinicians, such as hyperlipidemias, cardiomyopathies, and channelopathies. We designed a streamlined workflow to facilitate the importation of clinical and genetic data into GMG by potential collaborators. Crowdsourcing through GMG will expand the clinical and genetic content over time.

Long-term plans to improve the usability of GMG include a system to alert users when a similar variant is entered into the application; an optimized interface for mobile devices; multilingual support; and, in collaboration with the UTHealth Houston medical education team, enhancements for visually impaired users such as customizable colors and fonts, descriptive text, and screen readers. We acknowledge that the current version of GMG is not optimized for patients from diverse sociodemographic populations. We will collect demographic and survey data from patients to increase the relevance and clarity of GMG output for users with lower health literacy. We will expand and further automate data uploads into GMG as more users and clinical experts contribute data. To increase access to GMG, we will also seek collaborations with Epic Systems Corporation and other health care software companies to integrate GMG content into electronic health records.

GMG is intended to expand access to genetic information in an era when most cardiovascular genetic tests are not supervised by genetic professionals. GMG can prevent deaths due to genetic cardiovascular diseases by promoting evidence-based guidelines for gene-based treatment and familial testing. Additional studies are needed to evaluate how the implementation of GMG can change clinician decision-making and increase patient insight into heritable cardiovascular diseases.

## Supplementary material

10.2196/55903 Multimedia Appendix 1 Sample test report and questionnaire distributed to users for efficacy test of the Genomic Medicine Guidance application.

## Abbreviations

ACC: American College of Cardiology
AHA: American Heart Association
CSS: Cascading Style Sheets
GMG: Genomic Medicine Guidance
JS: JavaScript
TAD: thoracic aortic aneurysms and dissections
UTHealth Houston: The University of Texas Health Science Center at Houston
