# Supplementary material for: Development and Assessment of a Point-of-Care Application (Genomic Medicine Guidance) for Heritable Thoracic Aortic Disease
Source: JMIRx Med. 2024 Oct 8;5:e55903. doi: 10.2196/55903 (PMC11478091; doi:10.2196/55903)
Supplement: Multimedia Appendix 1 [file xmed-v5-e55903-s001.docx]

Sample Test Report provided for the Efficacy Test

**Survey Questions For Efficacy Test**

**Question 1.** By age 25, the estimated penetrance of (probability of having) aortic events for individuals who have this genetic variant is:

1. 100%
2. 50%
3. 25%
4. 0%
5. Can’t tell

**Question 2.** Individuals with this variant may have several distinctive features. Please select all choices that apply:

1. GI hypomotility and constipation
2. Lens dislocation
3. Mydriasis (fixed dilated pupils)
4. Bifid uvula
5. Patent ductus arteriosus
6. All of these features

**Question 3.** Which of the following are true about elective aortic repair in patients with this variant? Select all choices that apply:

1. The diameter threshold is 5.0 cm.
2. If the aorta is dilating rapidly, the diameter threshold is 4.2 cm.
3. The diameter threshold is 4.5 cm.
4. If the aorta is dilating rapidly, the diameter threshold is 4.5 cm.
5. The diameter threshold is the smallest known diameter of anyone in the family who had an aortic dissection at the time when they dissected.

**Question 4.** Refer back to the genetic test report. In counseling patients about the two results in this report, what should you tell them? Select all choices that apply:

1. The patient’s disease is entirely caused by the pathogenic variant.
2. The patient’s disease may be caused by the VUS, but additional testing is needed to be certain.
3. The patient’s disease is probably worse because they have two variants in two disease genes.
4. The patient should check back with the testing team in 3-5 years to find out if updated information is available for their test results.
5. No clinical decisions should be made based on the VUS result.
6. You should not tell them any of these things.

**Question 5.** You are seeing an asymptomatic 18-year-old who has this genetic variant. Which of the following tests are indicated? Select all that apply

1. Cerebral angiogram
2. Nuclear stress test
3. Optho exam
4. Bladder ultrasound
5. All of these tests
